# Management of horizontal duodenal perforation: a report of three cases and review of literature

**DOI:** 10.1186/s40792-017-0397-9

**Published:** 2017-12-01

**Authors:** Pramod Nepal, Kosei Maemura, Yuko Mataki, Hiroshi Kurahara, Yota Kawasaki, Kiyokazu Hiwatashi, Satoshi Iino, Masahiko Sakoda, Takaaki Arigami, Sumiya Ishigami, Hiroyuki Shinchi, Shoji Natsugoe

**Affiliations:** 1 Department of Digestive Surgery, Breast and Thyroid Surgery, Kagoshima University Graduate School of Medical and Dental Sciences, Kagoshima, Japan; 2 Kagoshima University Graduate School of Health Sciences, Kagoshima, Japan

**Keywords:** Horizontal duodenal perforation, Abdominal trauma, ERCP-related perforation

## Abstract

**Background:**

Perforation of the horizontal duodenum is very rare due to the presence in retroperitoneal space. It depicts an unusual clinical picture and is difficult to diagnose, leading to increased morbidity and mortality. The treatment strategies are usually varied and based on small series of cases, literature reviews, and expert opinions.

**Case presentation:**

Here, we presented three cases of horizontal duodenal perforation in three different clinical processes. The first case, a 30-year-old male patient, presented with abdominal pain and hematemesis after experiencing a physical assault on the previous day. Computed tomography (CT) scan showed rupture of the horizontal duodenum. It was repaired by side-to-side duodenojejunostomy. Postoperatively, he had anastomotic leakage, disseminated intravascular coagulation, and pulmonary failure and recovered after a long hospital stay. The second case, an 81-year-old female, had duodenal perforation with endoscopic coagulation of the bleeding diverticulum. Segmental resection of the duodenum and side-to-side duodenojejunostomy were performed. Postoperatively, there was slight anastomotic leakage, but surgical intervention was not needed. The third case, an 89-year-old female, was a patient with obstructive jaundice due to pancreas head carcinoma, who developed perforation of the horizontal duodenum during endoscopic retrograde cholangiopancreatography (ERCP). After unsuccessful conservative management, duodenojejunostomy at the perforated site and gastric bypass were performed. The postoperative course was uneventful.

**Conclusion:**

Early suspicion and investigation is necessary for cases of abdominal injuries. CT scan is the investigation of choice. The management options should be based on the clinical condition of the patient, comorbidities, surgical expertise, existing guidelines, and available resources.

## Background

Injury to the horizontal part of the duodenum is relatively rare because of the presence of retroperitoneal space [1]. It extends from the fourth lumbar vertebra to the level of the aorta. Since this part is adjacent to the spine, it is often affected by trauma [2]. The clinical examination result is initially negative and may appear only when the duodenal contents enter the peritoneal cavity. Thus, the early diagnosis of duodenal injury is very critical, resulting in delayed diagnosis, which contributes to the development of severe septic and inflammatory complications [3]. Endoscopic retrograde cholangiopancreatography (ERCP)- and endoscopy-related iatrogenic perforations are also very rare, and hence, the management options are varied and guided by results of small series of cases, literature reviews, and expert opinions [4, 5]. Here, we discuss three different cases of perforation of the horizontal duodenum and the variation in their management.

## Case presentation

### Case 1

**Fig. 1:**
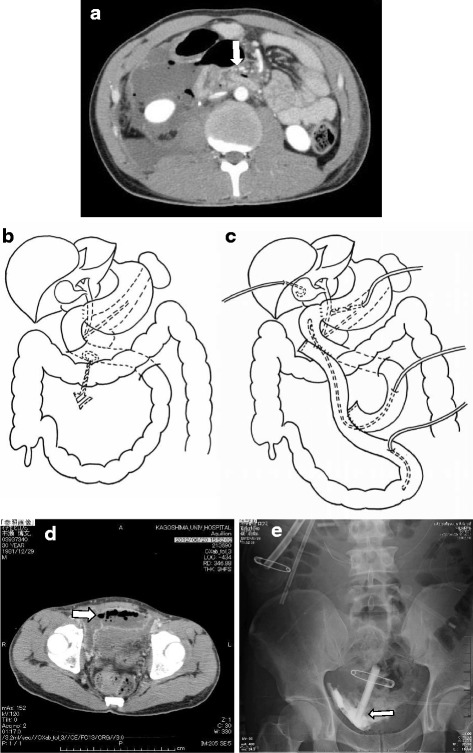
**a** CT scan showing rupture of the horizontal duodenum, free air, and perirenal fluid collection. **b** Perforation in the horizontal duodenum. **c** Side-to-side duodenojejunostomy was performed with decompressive gastrostomy and duodenostomy, feeding jejunostomy, and percutaneous transhepatic bile drainage. **d** Collection in the pelvic cavity 1 month post-surgery. **e** Plain radiogram showing abscess collection and percutaneous drainage tube in pelvic cavity

A 30-year-old male patient experienced physical assault while he was under alcohol influence, resulting in bruises all over his body. The next day, he developed abdominal pain and hematemesis for which he went to a nearby hospital where abdominal computed tomography (CT) showed free air in the abdominal cavity. He was then referred to our hospital for further management. The preoperative investigations are presented in Table 1. Consecutive CT scan of the abdomen showed increased tendency of free air, rupture of the horizontal duodenal wall, perirenal abscess collection, and hematoma in segment IV of the liver (Fig. 1a). Emergency laparotomy was performed, which showed type Ia injury of segment IV of the liver and retroperitoneal collection of bile mixed fluid. Complete dissection and kocherization of duodenum was performed, exposing a perforation in the horizontal duodenum involving half of its circumference (Fig. 1b). Side-to-side duodenojejunostomy was performed at the perforated area, along with decompressive gastrostomy, decompressive duodenostomy, feeding jejunostomy, and percutaneous transhepatic gall bladder drainage (Fig. 1c). A drain was kept at the anastomotic site, Douglas cavity, and paracolic gutter, and the abdomen was closed. Postoperatively, the patient had anastomotic leakage which did not need any intervention, disseminated intravascular coagulation, and pulmonary failure for which he was supported with artificial ventilation. One month post-surgery, he developed pelvic abscess which was not connected to abdominal cavity (Fig. 1d). It was successfully resolved after drainage with ultrasound guided percutaneous insertion of Penrose drain catheter (Fig. 1e). He had a full recovery after a long hospital stay of 126 days (Table 2).

**Table 1:** Preoperative laboratory investigations

Investigations	Case 1	Case 2	Case 3
Hemoglobin (g/dl)	19.2	5.5	8.2
Leucocyte count/μL	2690	3710	8380
Platelets/μL	180,000	112,000	335,000
Total bilirubin/direct bilirubin (mg/dl)	2/0.9	0.7/0.3	18/13.5
AST (U/L)	77	16	87
ALT (U/L)	61	14	93
Amylase (U/L)	556	64	341
CRP (mg/dl)	37.6	49.6	5.4
BUN (mg/dl)	27.5	64.6	27.9
Creatinine (mg/dl)	1.05	1.02	1.05
Total protein/albumin (g/dl)	6.1/3.3	4.7/1.7	5.6/2.3
PT (%)	72%	77%	86%
APTT (seconds)	31.6	34.9	29.7
Fibrinogen (mg/dl)	1109	838	5.1
D-dimer (μg/dl)	5.1	2.9	1.2

**Table 2:** Perioperative and postoperative outcomes

Variables	Case 1	Case 2	Case 3
Operative time	6 h 7 min	5 h 17 min	4 h 59 min
Blood loss (ml)	1630	1508	535
Postoperative complication (Clavien-Dindo grading)	Grade IV (pulmonary failure, DIC)	Grade II (slight anastomotic leakage)	None
Hospital stay (days)	126	33	15

### Case 2

**Fig. 2:**
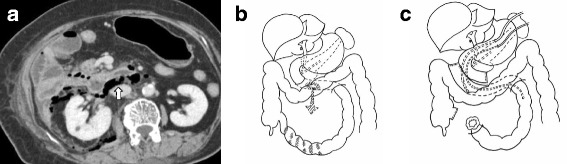
**a** CT scan showing a diverticulum in the horizontal duodenum and retroperitoneal free air. **b** Perforation site in the horizontal duodenum. **c** Side-to-side duodenojejunostomy was performed along with resection of the necrotic terminal ileum and stoma formation

An 81-year-old female patient with past history of abdominal surgery (details unknown) had presented with anorexia, general fatigue, and anemia. Upper gastrointestinal endoscopy revealed a diverticulum in the horizontal portion of the duodenum and bleeding from it. Endoscopic coagulation of bleeding was done, and she was kept nil per os for 2 days. The patient developed abdominal pain on the third postoperative day, with signs of peritonitis. Abdominal CT scan showed a diverticulum in the third part of the duodenum with perforation, retroperitoneal free air, and poor enhancement of the terminal ileum (Fig. 2a). She was referred to our hospital for further management during which she was in an anemic and severe inflammatory state (Table 1). Emergency laparotomy revealed severe intraabdominal adhesions and retroperitoneal abscess formation. Extensive dissection of adhesions and complete mobilization of the duodenum showed a diverticulum and perforation in the posterior wall of the third part of the duodenum and approximately 50 cm of necrotic terminal ileum (suspected non-occlusive mesenteric ischemia), shown in Fig. 2b. The duodenum was resected proximally at the end of the second part and distally 15 cm from the attachment of the ligament of Treitz followed by side-side duodenojejunostomy. Forty-five centimeters of the ileum, 5 cm proximal from the ileocolic junction, was resected, and ileostomy with a proximal end was done (Fig. 2c). The abdomen was closed after keeping a feeding tube and tube duodenostomy along with a drain in the Douglas cavity, subhepatic, and left subphrenic region. Postoperatively, although she had slight anastomotic leakage, any intervention was not required (Table 2).

### Case 3

**Fig. 3:**
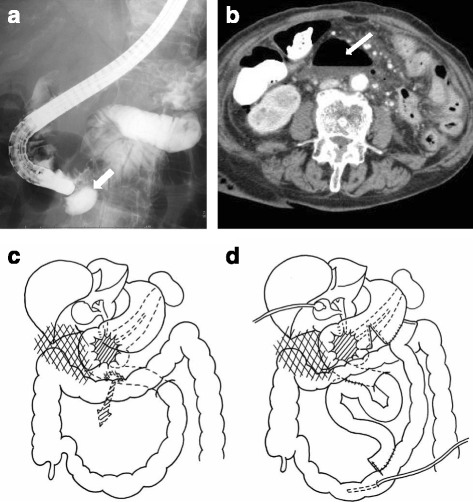
**a** Fluoroscopy shows extraintestinal leakage of the contrast medium. **b** CT scan showing rupture in the horizontal duodenum and abdominal free air. **c** Perforation site in the horizontal duodenum. **d** Side-to-side duodenojejunostomy with gastric bypass was performed along with resection of the necrotic terminal ileum and stoma formation

An 89-year-old female patient, living in a retirement home, developed loss of appetite and jaundice. She was taken to a nearby hospital. Her CT scan showed carcinoma of the pancreas head, stage III (T3N0M0). She was referred to our hospital with diagnosis of obstructive jaundice with renal dysfunction with stage III pancreas head carcinoma for further management. Also, the laboratory data showed slight elevation of inflammatory responses and severe hyperbilirubinemia (Table 1). She was subjected to ERCP, during which duodenal perforation was suspected. Intraoperative fluoroscopy showed extraintestinal leakage (Fig. 3a). Initially, she was managed conservatively with nil per os, intravenous fluids, and antibiotics. On second post-ERCP day, she developed signs of peritonitis. Abdominal CT scan showed perforation at the horizontal duodenum (Fig. 3b). Emergency laparoscopic surgery was planned, but due to extensive intraabdominal adhesions, laparoscopy was converted to laparotomy. Around 10 cm of pus and air collection was found in the root of the mesojejunum along with a 2-cm perforation at the horizontal duodenum which was adjacent to the tumor site (Fig. 3c). Side-to-side duodenojejunostomy and gastric bypass with gastrojejunostomy and jejunojejunostomy was done. Biliary drainage was maintained with a balloon catheter, and feeding jejunostomy was done (Fig. 3d). Peritoneal lavage was performed, pelvic and anastomosis site drain was kept, and the abdomen was closed. The postoperative course was uneventful (Table 2).

## Discussion

It has been reported that the causes of horizontal duodenal perforation are trauma or iatrogenic injury due to ERCP mainly. Duodenal injury is present, on average, in 3.7–5% of abdominal injuries and may be due to either blunt trauma of the abdomen or penetrating injuries [6]. Though incidence of iatrogenic injuries during upper gastrointestinal endoscopy alone is extremely rare, it is significantly higher in ERCP, estimated to be between 0.4 and 1% [5, 7].

Chest and erect abdominal radiography and ultrasonography are not of diagnostic value, and the modality of choice is CT scan with both oral and intravenous contrast media [2, 3]. Factors like anatomical location of the injury, type and extent of injury, associated injuries to other structures and organs, and time of surgery determine the type of surgical options and their outcome [8]. The American Association for the Surgery of Trauma has suggested the Organ Injury Scale [9], but the grading may not always dictate the management [1]. Likewise, several authors have proposed the immediate surgical repair of ERCP-related duodenal perforations [10].

The surgical options available for repairing perforation are simple repair (duodenorrhaphy), resection and anastomosis, repair and decompressive enterostomy, serosal or mucosal patch, pyloric exclusion, duodenal diverticulization, and pancreaticoduodenectomy [11]. Majority of perforations can be managed by simple repair or resection and anastomosis. Duodenal diverticulization and pancreaticoduodenectomy are rarely required [3, 11]. In our first case, blunt trauma to the abdomen resulted in perforation of approximately half of the circumference of the duodenum (grade III injury). Side-to-side duodenojejunostomy was preferred and a safe method for this case. For the protection of the suture line by decompression of the anastomotic site, we made a tube gastrostomy, tube duodenostomy, and percutaneous transhepatic drainage.

Damage control surgery consisting of an initial abbreviated surgery to control bleeding and contamination, followed by correction of hypothermia, coagulopathy, and acidosis in the critical care unit and timely re-exploration, has promising outcomes in the management of patients with critical trauma [12]. Endoscopic closure by endoclips is found to be a safe, feasible, and effective technique for the treatment of ERCP-related duodenal perforation [13].

The injury in the second case was due to therapeutic endoscopic coagulation which was diagnosed in the third postoperative day. Due to the presence of a diverticulum in the third part, unhealthy wound margins, and adjacent superior mesenteric artery, segmental resection of the duodenum was effective. It was important to make an anastomosis at the healthy descending duodenum. In the third case, injury to the third part of the duodenum during ERCP was most probably the result of a duodenal abnormality secondary to neoplasm. We preferred non-surgical management initially because of the elderly patient’s poor general condition and associated comorbidities, which ultimately led to a failure to respond. Due to the presence of a tumor site close to the perforation area, narrowing of the descending duodenum, and adhesions, we opted for duodenojejunostomy and gastric bypass by gastrojejunostomy and jejunojejunostomy.

An association of duodenal rupture with other intraabdominal organ injuries and leakage of a large volume of pancreatic and biliary secretion causes severe sepsis, contributing to its significant mortality (6 to 25%) and morbidity (30 to 60%) [3]. Among our cases, the first case had a significant morbidity (Clavien-Dindo grade IV [14]), and the second case had grade II complications. However, the postoperative period of the third case was uneventful, and there was no mortality among the cases. All of the emergency surgeries were done in a single setting, and we did not feel the need of damage control surgery in our cases. We also recommend pyloric exclusion as an alternative approach in case of suspicion of possible wound leakage.

## Conclusions

Duodenal injuries require a high level of suspicion and careful examination. The management options for such cases are variable and depend upon multiple factors and should be guided by existing literature reviews, clinical status of patients, presence of coexisting diseases, clinical judgment and expertise of the attending physician, and availability of resources.

## Abbreviations

CT: Computed tomography
ERCP: Endoscopic retrograde cholangiopancreatography
